# Supplementary material for: The eyes don’t have it: Eye movements are unlikely to reflect refreshing in working memory
Source: PLoS One. 2022 Jul 14;17(7):e0271116. doi: 10.1371/journal.pone.0271116 (PMC9282440; doi:10.1371/journal.pone.0271116)
Supplement: Attachment — Submitted filename: coverletter_v2.0_response2reviewers.docx [file pone.0271116.s001.docx]

Vanessa M. Loaiza

Department of Psychology

University of Essex

Wivenhoe Park

Colchester

CO7 9JH

United Kingdom

Telephone: +44 (0)1206 873779

Email: [v.loaiza@essex.ac.uk](mailto:v.loaiza@essex.ac.uk)

Colchester, 26 May 2022

David K Sewell

Associate Editor, PLOS One

Dear Dr. Sewell,

Attached is the revision of unmasked manuscript PONE-D-22-00865 entitled, *Tracking eye movements during spontaneous and instructed refreshing in working memory,* to be considered for publication as a regular article in PLOS One. The manuscript is original, and the data are not published or being considered elsewhere for publication.

Data collection of Experiment 2 was supported by a grant from the Swiss National Science Foundation to A. S. Souza (project 1000019_169302). The funders had no role in the study design, data collection and analysis, decision to publish, or preparation of the manuscript.

In this letter, we have responded to each of the comments in detail. Comments were enumerated for clarity (i.e., E = editor, R = reviewer, e.g., R1.1) and our responses follow each comment in boldface. Any changes accompanying the responses to the comments were noted both in the response with the page number and in the manuscript using red font. We also revised our OSF page (which contains data, analysis scripts, and materials) to reflect any analysis changes while also retaining the original analyses to preserve transparency and visibility of this work.

We were grateful to the overall positive feedback received, and the issues that arose largely concerned clarification about the methods/analyses as well as some organizational issues. We believe that we have addressed all these constructive comments that we received, which we briefly summarize here:

1. First, we clarified issues about the methods, such as those regarding differences in the eye-tracking methods between the two experiments (R1.21), the presentation and timing of the refreshing cues (E1.4, R1.12, R1.13), the methods of drawing inferences (R1.17), and how the methods of the experiments address the research questions (R2.4).
2. Second, we clarified issues about the analyses and results: We resolved the issue regarding the results matching between the text and what is now Figure 6 regarding Research Question 3 (E.1, R1.6), thereby resulting in a stronger conclusion (R2.6). We also followed the advice regarding the analysis presented in what is now Figure 4A to rescale the fixations (E.3, R1.7, R1.8), which served to resolve the apparent discrepancy between two of the analyses reported previously. We also provided more information about the models used in the analyses (E.5, E.6, R.10) and considered the recommended alternative measures in the analyses (E.7, R1.9). Finally, we revised various aspects of the results to make them clearer and easier to understand (R1.3, R1.11, R1.15, R1.16, R1.18).
3. Finally, we structured the results section so that it was easier to follow and understand which experiment and measure was addressed for each reported analysis under different subsections of the research questions (E.2, R2.3). This allows a compromise of retaining the original research-question-driven structure of the manuscript while still being very clear how each experiment and analysis addressed the relevant research question.

In summary, we believe the revised manuscript addresses each of your and the reviewers’ concerns and is substantially improved thanks to these thoughtful comments. We hope that you and the reviewers feel more satisfied with the conclusions of the manuscript, and we are grateful for this constructive feedback and thorough assessment of our work. We appreciate the opportunity to revise the manuscript, and we look forward to hearing from you.

Sincerely,

Vanessa M. Loaiza

Alessandra S. Souza

**Editor’s comments**

**E.1:**

Thank you for submitting your manuscript to PLOS ONE (PONE-D-22-00865). I have now received two expert reviews on your submission, and I have also read through it myself. The first review is a joint review signed by Kyle Hardman and Nelson Cowan. The second reviewer has chosen to remain anonymous.

As you’ll see, both reviewers found the manuscript to be clearly written and the studies to be well-designed. I fully agree with their assessments in this regard. Further, the work is on a timely and theoretically interesting topic. There are, however, serious concerns about the interpretation of the analyses, which I share. The main issues are as follows:

1 – Conclusions regarding Research Question 3 do not obviously follow from the data. Reviewer 2 highlights the non-monotonic pattern of effects of fixations on precision (i.e., positive for 1 cue, but null for 2). Reviewer 1 notes there are some issues with coherence of effects across experiments, and identifies apparent discrepancies between the summaries of the analyses and the plots of the effects.

**Response:**

**Thank you for these positive comments. As we explain in response to Reviewer 1 (R1.6), two issues caused the apparent discrepancy regarding Research Question 3: First, the plot mistakenly showed the cue frequency x fixations interaction for the cue duration of 0.5s (which was our reference condition). Figure 6 now shows the full three-way design for completeness. Second, we have properly followed up the interaction using the emmeans package in R as recommended for the brms package, and so the revised text (pages 22-23) now matches what is displayed in Figure 6. We have also addressed any lingering issues of Reviewer 1 in response to their comments in the same thorough manner, explaining in detail with additional plots where necessary. We apologize for the confusion, but thank you for pointing this out so that we can be certain that the reported results are accurate and presented coherently.**

------

**E.2:**

2 – It is sometimes difficult to fully the results due to how this section has been organized around research questions rather than individual experiments. Reviewer 1 notes that some of the analysis details are only implied in places. I agree that more specific information should be included throughout to improve readability. Reviewer 2 comments similarly that it is not always clear which experiment is being referred to with regards to Experiments 1a and 2 (i.e., are they being individuated or lumped together). While I appreciate the purpose of organizing the results as you did, I found it quite difficult to keep abreast of exactly what was being analyzed at any given moment. It may be somewhat less efficient to analyze the study results individually, I think it may be beneficial to assist the reader in following what was done. I am open to keeping the current structure, and it may be the case that more careful attention to detail will solve these issues, but my overall sense is that an experiment-by-experiment structure would be clearer.

**Response:**

**Thanks to these helpful comments, our revised manuscript is now more explicit about the analysis details and we have restructured the results to improve coherence. As we explain further on (R1.10), we have clarified various details about our analyses, such as more information regarding the mixed effects approach that we took and the models that we used (pages 15-17). Furthermore, to address the issue of organization (R2.3), the revised manuscript now more clearly separates the different analyses of each experiment into separate subsections within the three different research questions. We have also reiterated throughout each subsection which experiment and measure is being assessed, with stronger introductions to each analysis and with clearer reference to the figures as well. These changes can be seen throughout the results section on pages 17-23. We hope this makes it clear which experiment and analysis are considered while also retaining how those experiments/analyses address each research question.**

------

**E.3:**

3 – There’s a lack of clarity surrounding the data presented in Figure 3. Although you do attempt to address the apparent inconsistency in the results across the two panels in the figure, I didn’t find the explanation especially convincing. Reviewer 1 suggests a potential way to resolve this issue and more meaningfully present the fixation rate results. I think this is an excellent suggestion and encourage you to consider pursuing a “scaled” analysis of these data.

**Response:**

**We agree that this was an excellent suggestion from Reviewer 1. As we explain further below in response to comment R1.7 and R1.8, scaling the fixation rate to the cued locations according to the number of items in the array that were not cued, cued once, or cued twice presented a much clearer and comprehensible result that was consistent with the analysis of the fixations to the target. We have revised what is now Figure 4A and the text on pages 19-20 and 24-25.**

------

**E.4:**

In addition to these three main issues, I add a few smaller comments based on my reading of the manuscript below.

4 – For the ABA trials in Experiment 1b, did the cues always alternate between different items, or could the same item be cued twice in succession? That is, did the “ABA” trials include “AAB” and “BAA” sequences too?

**Response:**

**We have clarified this on page 12, stating that A-B-C and A-B-A “were the only two possible sequences of refreshing cues; thus, the cues always alternated between different items, and no items were cued twice in succession.”**

------

**E.5:**

5 – On page 15, you note that responses were centered on the target value. However, no data or analysis are presented to corroborate this. Can you include a plot of the aggregated data and/or could you verify the centering by fitting a von Mises with a freely estimated mean and show (for example) that the credible interval overlaps 0?

**Response:**

**On pages 15-16 we now refer to Figure 2 that includes density plots for both fixation rate and signed recall error (in radians) for all the experiments. The plot of recall error in particular shows more clearly how responses were centered on 0 with long tails, as is typical in a von Mises distribution. We also conducted analyses wherein the mean is freely estimated; as we note in footnote 5 on pages 16-17, there were no credible effects on the mean. These analyses can be found on the OSF.**

------

**E.6:**

6 – The reporting of the analyses for Research Question 1 was ambiguous with regards to what parameter was being affected by the various factors outlined in Table 1. You mention that fixation rate was assumed to be ex-Gaussian distributed. Do the reported estimates and effects pertain to the mean, or are the variance and tau parameters also affected? This needs to be more clearly presented.

**Response:**

**We have now clarified on pages 15-16 that the fixation rate analyses for the experiments that are summarized in Table 1 are effects on the mean of the ex-Gaussian distribution, whereas we did not apply their effects to sigma and tau (called “beta” in brms).**

------

**E.7:**

7 – This point is mentioned in detail by Reviewer 1, but I had similar thoughts regarding fixation number vs. duration. For example, are fixations to cued locations fewer in number, but longer lasting? Analysis of fixation duration seems more important than just number of fixations for matters related to attention, as noted by Reviewer 1. There is also some precedent in the categorization literature using fixation duration (or proportion of trial time fixating an item/feature) as an assay of attention (e.g., Rehder & Hoffman, 2005, Cognitive Psychology).

Rehder, B., & Hoffman, A. B. (2005). Eyetracking and selective attention in category learning. Cognitive Psychology, 51(1), 1–41. <https://doi.org/10.1016/j.cogpsych.2004.11.001>

**Response:**

**We agree that this is an important point to consider. As we explain in further detail in response to R1.9 and in footnotes on pages 16-17, fixation rate/frequency (used in the manuscript) and fixation duration were strongly correlated across experiments, and thus multicollinearity prevented us from including fixation duration alongside fixation frequency in the analyses for research questions 2 and 3. Moreover, the pattern of results was the same regardless of using fixation rate/frequency or fixation duration, which is not surprising given how strongly correlated these variables were. We have updated the results on the OSF to include analyses using fixation duration instead so that interested readers can verify this.**

**Reviewer 1’s comments**

**R1.1:**

In this study, the authors examined whether fixations to memoranda locations during working memory maintenance could be interpreted as reflective of a memory refreshing process. The task was working memory for colors in which continuously varying colors were presented at differentiated locations. Eye tracking was used during the task to measure eye fixations to memoranda locations.

This study had three main questions and the evidence related to each question is evaluated below. The answers to two questions were reasonable, but there were serious issues with the answer to the third question. In addition, there were some general concerns with the study that are explained in sections below.

Overall, the study appears to be well conducted and the method is suited to answering the research questions. The issues are mostly related to analysis. As such, the recommendation is that the authors submit a major revision that addresses the identified issues.

**Response:**

**Thank you very much for these positive comments and your very thoughtful and helpful feedback. We believe that the manuscript is much improved thanks to your constructive advice.**

------

**R1.2:**

Research Questions:

Q1: Do participants look at locations of previously presented memoranda? (i.e., the looking-at-nothing effect.)

For Q1, the authors found evidence that participants look at the locations of memoranda during the RI in Exp1A (Fig2). However, in Exp2 when location placeholders were absent, participants had little preference for fixating memoranda locations. In Exp2, fixating on memoranda locations required eye movement to a particular region of the screen, whereas in Exp1A, memoranda locations were in all directions from the initial fixation. Thus, memoranda location fixations in Exp2 are more meaningful than in Exp1A. Based on this evidence, the authors conclusion that participants do not purposefully look-at-nothing is reasonable.

A subcomponent of Q1 is the question of whether cues to memoranda locations can cause fixations (cued looking-at-nothing; Exp1B). It seems like the cues do cause looking-at-nothing (Fig3), but the data in Fig3 could be analyzed better as explained in the section Experiment 1B Fixation Rate Scaling below. Spontaneous looking-at-nothing is a much better indication of a refreshing process than cued looking-at-nothing which may be more reflective of participants doing what they are told to do.

The best answer to Q1 comes from Exp2, which provides a reasonable answer.

**Response:**

**Thank you for this synopsis. We have addressed the scaling issue further on in response to R1.7.**

------

**R1.3:**

Q2: Does spontaneous looking-at-nothing affect recall precision? (Exp1A and Exp2)

For Q2, the authors found evidence against spontaneous looking-at-nothing providing a benefit to recall precision (Fig4). Other than an apparently spurious interaction, there was no clear evidence that looking at memoranda locations provided a benefit. The effect of fixations on precision in Exp1A neared significance, which could explain why other authors find such an effect (discussed more later).

We assume that this analysis examined fixations during the retention interval only, and not fixations during encoding, but we could not find a clear statement to that effect. In revision, adding such a statement would be helpful.

**Response:**

**We have made it clearer on page 16 that the analyses for research questions 2 and 3 pertain to fixations during the retention interval (RI) only.**

------

**R1.4:**

The authors provide a reasonable answer to Q2, but there is the limitation that fixation duration was not included in the analyses (discussed more later).

**Response:**

**We have addressed the issue of fixation duration further on in R1.9.**

------

**R1.5:**

Q3: Does looking-at-nothing when cued to do so affect recall precision? (Exp1B)

More refreshing cues improved precision (Fig5B), but that is not reflective of looking-at-nothing per se: When cued to a particular memorandum, people may reallocate WM resources to the cued item without fixating the cued location.

More specific to Q3, a main effect of fixations on precision was found and an interaction between the number of cues and fixations on precision was also found (Fig5A and C). There are two issues of different types with these results.

Issue 1: The main effect of fixations in Exp1B goes against the results of Exp1A which found no effect of fixations. The effect in Exp1A is in the same direction as Exp1B and of similar magnitude, but the significance tests go in different directions (Exp1A not significant, Exp1B significant). Maybe there is a true effect of fixations on accuracy that was not quite picked up in Exp1A, which would bring part of the answer to Q2 into doubt (although Exp2 provides the better evidence, so Q2 still has a reasonable answer). The authors might benefit from comparing the Exp1A and Exp1B results in terms of similar magnitudes rather than relying on hypothesis testing logic alone.

**Response:**

**Indeed, the overall effect of fixations was numerically and directionally similar between Experiment 1A (estimate = 0.51 [-0.12, 1.15]) and Experiment 1B (estimate = 0.59 [0.11, 1.11]). However, as evident in the credibility intervals reported here and in what are now Figures 5 and 6, the range of the variability was simply greater in Experiment 1A than in Experiment 1B. Given that Experiment 2 was designed to address lingering issues of Experiment 1A, we agree that the lack of credible effect in Experiment 1A is probably the true state of affairs and may reflect that fixations in spontaneous conditions are not functional. We have added more discussion on this point on page 24.**

------

**R1.6:**

Issue 2: The interaction as plotted in Fig5C shows that fixations provide the most benefit to items that were cued less often. However, the figure seems inconsistent with estimates presented in the text (P20): “This interaction hinted at a decreasing sensitivity of kappa to fixation frequency as the number of refreshing cues increased: Follow-up analyses showed that the effect of fixations was not credible for 2 refreshing cues (estimate = 0.06 [-0.20, 0.33]), positive and credible for 1 refreshing cue (estimate = 0.50 [0.11, 0.90]), and positive but not credible for 0 refreshing cues (estimate = 0.27 [-0.22, 0.77]; see Figure 5C).” The sign for 2 cues disagrees between the text (small positive) and figure (small negative). In the text, the magnitude of the effect on fixations on kappa for 1 cue is larger than 0 cues, but the figure shows the reverse (the slope for 0 cues is steeper than 1 cue). The analyses for this interaction need to be double-checked.

The authors do not adequately answer Q3, but it may be possible to address the issues in revision.

**Response:**

**Thank you for pointing this out. Two issues caused the discrepancy: First, the plot mistakenly showed the cue frequency x fixations interaction for the cue duration of 0.5s (which was our reference condition). Figure 6B now shows the full three-way design for completeness. Second, we had followed up the cue frequency x fixations interaction by conducting separate models to examine the effect of fixations for each cue (0, 1, and 2), but we have since learned that this is not the proper way to follow up interactions in brms. Instead, in the current version we used the emmeans package in R to follow up the effect of fixations at levels of the other interacting independent variable (in this case, the number of cues). We have updated the results on the OSF and on pages 22-23 (and also for following up the interaction in Experiment 1A between RI and fixations on page 21). As we now report, the effect of fixations was credible for 0 refreshing cues (estimate = 0.51 [0.09, 0.96]), smaller but still credible for 1 refreshing cue (estimate = 0.31 [0.02, 0.59]), and further smaller and not credible for 2 refreshing cues (estimate = 0.11 [-0.23, 0.42]). We apologize for the confusion, but thank you for pointing this out so that we can be certain that the reported results are accurate and presented coherently.**

------

**R1.7:**

Experiment 1B Fixation Rate Scaling

The pattern in Fig3A seems to be that cues cause participants to fixate 0-cued locations, but that doesn’t make sense and doesn’t agree with Fig3B. One way to address this issue is for the fixation rates in Fig3A to be scaled based on the proportion of items that are cued 0, 1, or 2 times. More explanation:

One trial type is ABC and the other ABA. On ABC trials there are three single cued items and N-3 uncued items. On ABA trials there are 1 double cued item, 1 single cued item, and N-2 uncued items. The authors say N is usually 5 or 6 and let’s imagine N=6 for sake of example. On a combination of 1 ABC trial and 1 ABA trial are 12 total items, and in terms of cues a total of 1 double cued, 4 single cued and 7 uncued items. Fixation rate, averaged (by eye) across cue durations, are approximately 4 for double cued, 4.5 for single cued, and 5 for uncued. Taking the number of items of each cue type into account, double cued items had a fixation rate of 4 for 1 item (ratio=4), single cued had 4.5 for 4 items (ratio=1.1), and uncued had 5 for 7 items (ratio=0.7). Looking at the ratios shows that participants are fixating cued items at a rate higher than would be expected given the number/proportion of items of each type (vs the assumption of random fixations), but Fig3A seems to suggest the opposite. It is possible to take the proportions of cued item types into account when making Panel A? Or make an additional panel taking proportions into account?

Fig3B seems to show the real effect better because the proportions of cue types (double, single, uncued) are “equalized” (in a sense) because there is only 1 target. Even if there are more uncued items than other types, only 1 of those can be selected as a target, so fixations to other nontarget uncued items are not counted in Panel B.

**Response:**

**We are very grateful for this recommended scaling approach. Indeed, we had trouble to make sense of these results in what was Figure 3A. Thanks to your recommendations, the results explained on pages 19-20 and displayed in what is now Figure 4A reflect fixation rate to the cued locations that is scaled according to the number of items in the array that were not cued, cued once, or cued twice. This was determined for each trial for each participant, given that the task was individually calibrated. Consequently, the results of the two approaches are now much more consistent and comprehensible.**

------

**R1.8:**

The authors attempt to explain the discrepancy between Fig3A and Fig3B on P18 in the paragraph starting “At first glance, the positive effect of cue frequency in this second analysis appears to conflict with the negative effect in the first analysis.” Taking the cue type proportions into account is a better explanation than the one given in this paragraph.

Also, in the discussion P21: “When their attention was cued to refresh the memoranda (Experiment 1B), fixations occurred most frequently to non-cued locations.” While this statement is true, it is misleading because it doesn’t take the cue type proportions into account.

(To be clear: the analyses of recall precision, like in Fig5, should not be affected by the concerns about fixation rate scaling.)

**Response:**

**Given the recommended scaling that we carried out to address the previous comment R1.9, we have removed this paragraph on pages 19-20 and revised the discussion on what is now pages 24-25. Thank you once again for this helpful advice, it was just what we needed to make better sense of these results.**

------

**R1.9:**

Analyzing Fixation Duration

No estimate of the duration of a look was provided. The dependent variable for eye movements was “looking rate” defined as fixations per second, but fixation rate doesn’t take into account the total duration of looking at target versus non-target locations. For example, suppose each look to a target location lasts three times as long as each random look elsewhere. That would be an indication of preferential looking at a target location that is not picked up by the looking rate measure. It is not implausible that meaningful looks would last longer than non-meaningful ones.

When it is stated that “fixation rate decreased with increasing cues to memoranda locations (estimate = -1.21 [-1.87,-0.54]), such that participants looked more often to never-cued locations compared to once or twice-cued locations (see Figure 3A)” maybe that counterintuitive finding occurs because participants are looking at cued locations longer with each look.

It is possible that fixation duration is reflective of resource allocation or trying to remember/reconstruct the item at a location. It could be that analyzing fixation duration along with fixation rate could provide a more complete understanding of the data. In revision, the authors should examine fixation duration. If it is a poor measure, the reason why it is a poor measure should be given.

**Response:**

**Given the scaling approach recommended and applied in response to R1.7 and R1.8, the previous “counterintuitive” result of fixation rate to the cued locations is no longer relevant. However, the point that fixation duration may be more important than rate or frequency is important to consider. We tried to capture the “time” element by using fixation rate that is adjusted according to the duration of the encoding and RI phases (as we now make more explicit on page 15), but this is obviously not the same thing as how long participants spend looking with each fixation.**

**We did initially wonder if fixation duration should be included or used instead in all of the reported analyses. If we were to use this measure, it would need to be a proportion (i.e., the proportion of time spent looking at the target(s) out of the total duration of detected fixations), analogous to fixation frequency. We could not use absolute duration of fixations because, as explained previously, the encoding and RI phases were not equivalent in their durations, and in Experiment 1, the RI and cues were presented for different durations. Thus, proportion is most appropriate.**

**However, we initially decided against using fixation duration because we observed across experiments that it was highly correlated with fixation rate and fixation frequency, as is evident in the figure below (*r*s ranging 0.80 to 0.92).**

**Thus, fixation duration would have a high degree of multicollinearity if it were included in the models alongside fixation rate or fixation frequency. However, it could be used instead of fixation rate (for research question 1) or fixation frequency (for research questions 2 and 3) as we reported in the manuscript.**

**For the first research question concerning looking-at-nothing, it would not be analytically appropriate to use fixation duration in the analysis given that the location categories sum to 1. However, the plot below that is similar to what is now Figure 3 shows the same pattern of results as those reported for fixation rate, which is again not surprising given the strong relationship between the two factors.**

**For the second and third questions, it may be that fixation duration is a more effective predictor of WM recall than fixation frequency, as hinted at this comment and a similar one from the Editor (E1.7). However, we again found that the results were consistent with the current reported results using fixation frequency. We have made all of this explicit in a footnote on page 15 that the interested reader can find the results of both analyses with either fixation frequency (as reported in the manuscript) or fixation duration on the OSF.**

------

**R1.10:**

Model choice and purpose of modeling

It isn’t clear what working memory model is being used. It sounds like a precision-only model in that nothing is mentioned about any parameters other than precision (kappa). Is the used model like the Bays and Husain (2008) model?

Given the array sizes that were used, it is possible that many participants were below capacity, so some of the observed effects on precision could have been due not to reduced precision per se, but fewer items being in working memory (e.g., changes in the Pm parameter in the Zhang and Luck 2008 model). Readers could interpret kappa as memory precision alone with forgetting accounted for by other parameters in the model. The authors should address this point of confusion about the model.

It seems like the model is used as a kind of data transformation and that the analyses could be performed on the raw data (degrees of response error) instead of kappa. The authors should provide a reason for using the model instead of raw data.

Bays, P.M., & Husain, M. (2008). Dynamic Shifts of Limited Working Memory Resources in Human Vision. Science, 321, 851-854.

**Response:**

**It is not a working memory model per se so much as a distribution family offered by brms that fits any data with a circular normal (i.e., von Mises) distribution. We used this distribution given the distribution of signed recall error (see the now-included Figure 2, bottom panels) and the mixed effects approach that we now justified more explicitly on pages 14-15. As we also explain in response to the editor (E.5) and as is evident in Figure 2, the mean of the responses was centered on 0, and thus the only parameter left in this distribution family is the kappa parameter. We have conducted additional analyses that can be found on the OSF wherein the effects were applied to the mean of the von Mises distribution instead of kappa. However, it is probably not surprising given Figure 2 that none of these analyses yielded credible results, even of effects that anyone would expect, like a negative effect of output order in Experiment 2. We have noted this in footnote 5 on pages 16-17.**

**We recognize that the language of using this analytic approach prompts one to think of working memory mixture models and other possible parameters besides precision. Although both authors have extensive experience fitting even hierarchical Bayesian mixture models with categorical independent variables, we are unfortunately not equipped to apply them with a continuous fixed effect of fixation frequency/duration. Hopefully including all the raw data on the OSF will allow more capable researchers to take on the challenge, and we have at least noted that this may be of interest for future study in the General Discussion on pages 24-25.**

**Finally, we did consider using the absolute value of the recall error in degrees as suggested here in this comment rather than the signed distances transformed into radians that we used. For the analysis of recall, we considered using an ex-Gaussian distribution to fit the data in brms. However, many of these models failed to converge, even with many more iterations per chain (i.e., 10,000 instead of 2,000). In the instances where the model did converge, the pattern of results was the same as the von Mises distribution. We have made note of this in the revised manuscript in footnote 5 on pages 16-17, and deposited all the results on the OSF for interested readers.**

------

**R1.11:**

Exp1B Minor Issues

The language Within Trials vs Across Trials in Fig3 is unclear. Presumably, the data are aggregated across all trials for both plots. Also, one trial does not affect another trial, so that is not what is meant by across trials.

**Response:**

**This within/across language was removed from what is now Figure 4 and from the text of the corresponding analyses on pages 19-21.**

------

**R1.12:**

Exp1B method should clearly state whether the cues were informative (increased probability of testing the cued locations vs random test location). In the discussion random testing is suggested: “… but in the current work the refreshing cues of Experiment 1B did not clearly indicate the test target as retro-cues do.”

**Response:**

**Thank you for catching this. On page 11, we have stated this more clearly: “Participants were also explicitly informed that cues did not predict which item would be tested.”**

------

**R1.13:**

About the Exp1B retention interval duration, the authors say on P20: “… presenting refreshing cues for a longer duration yielded worse precision.” Was the RI longer when cues were longer? The method isn’t clear on this point.

**Response:**

**Yes, the RI was longer when the cues were longer. This is now clarified on page 11: “Following the offset of the last refreshing cue, a final ISI of 0.5s preceded the test. Thus, the entire RI of Experiment 1B lasted either 2.5 or 4s depending on the cue duration, just as in Experiment 1A.” As a reminder, there was no effect of RI duration in Experiment 1A, and thus it seems unlikely that the negative effect of a longer cue duration is due to a mere effect of total RI duration, which we reiterate on page 22.**

------

**R1.14:**

Misc Minor Issues

The explanation on P13 the threshold radius for classifying fixations is confusing, possibly because the units are wrong. The confusing quotes: “This threshold was defined as a radius of 142°, which is the radius around 4 dots (…) before touching the area around the next dot …” “The threshold for looking at the center of the screen was a radius of 57.5° …” Do you mean the center was defined as a circle with a radius of some length? If so, a length in degrees would make me think visual angle, but 57 degrees of visual angle would be too big. Are the radii pixels rather than degrees?

**Response:**

**Thank you for catching this. It should indeed be pixels, not degrees, and we have revised this on pages 13 and 14.**

------

**R1.15:**

For figures showing parameter intervals (like Fig 5 Panel A): Can you make the vertical line at 0 a little thicker so it stands out better?

**Response:**

**We have made this change to the plots of the parameter intervals of what are now Figures 5 and 6.**

------

**R1.16:**

For Table 1, can you spell out what the factors are in the three-way interaction? The four factors are Phase, RI, Placeholders, and Location. RI is not present in Exp. 2 and Placeholders are not present in Exp1, so we guess that the 3-way is Phase and Location plus RI (Exp1) or Placeholders (Exp2). Is this right?

**Response:**

**Yes, this is correct. To retain the conciseness of the table, we have clarified this information in the note of Table 1 now on page 18.**

------

**R1.17:**

Bayes factors are mentioned on P7, but it seems like you use credible intervals and not Bayes factors.

**Response:**

**In our pre-registration of Experiment 1 we had assumed that we would use Bayes factors with the BayesFactor package, but we instead we used an analogous mixed effects approach in brms, and thus we used credible intervals. We have now clarified this on pages 7 and 14-15.**

------

**R1.18:**

The graphs of fixation rate should include something like “Rate (looks per second)” on the Y axis so the meaning of “rate” is immediately obvious.

**Response:**

**Thank you for this good suggestion. We have adjusted the y-axes of Figures 3 and 4 accordingly.**

------

**R1.19:**

Some relevant literature was omitted: e.g., Mall, J.T., Morey, C.C., Wolff, M.J. et al. Visual selective attention is equally functional for individuals with low and high working memory capacity: Evidence from accuracy and eye movements. Atten Percept Psychophys 76, 1998–2014 (2014). https://doi.org/10.3758/s13414-013-0610-2

**Response:**

**Thank you for drawing our attention to this paper. Its relevance seemed most pertinent to fixations toward placeholders, and so we have included it now on page 6.**

------

**R1.20:**

Further Questions

Was there a tendency for locations fixated during encoding to be fixated again during retention? If this answer is yes: Might the benefit (if any) of fixating a location during retention be an artifact of attention returning to stimuli fixated during encoding, which are presumably encoded better than non-fixated stimuli? Specifically, when modeling memory precision, if fixations to the target at encoding are included as a covariate, does that account for some of the effect of fixations during retention on precision? In Exp1B, can you make the effect of fixations on precision go away by including fixations at encoding as a covariate? These questions might be better answered by researchers who find a substantial looking-at-nothing benefit than the current authors who found some benefit only in Exp1B. There is no need to answer these questions in revision, but we think they are good questions.

**Response:**

**We agree that this is a great question and one that is addressable in our data given that we recorded eye movements during the entire trial. There were relatively small but still positive correlations between fixation frequency during encoding and the retention interval (rs = .28, .25, and .34 in Experiments 1A, 1B, and 2, respectively). So, participants sometimes fixated on similar locations between encoding and the retention interval (RI). We attempted to address this by including encoding fixations in the reported models, but these models failed to converge. We can at least cursorily address the issue by plotting recall error (i.e., the distance between the target and the response in degrees) as a function of whether participants fixated on the target during encoding (no/yes) and/or the RI (no/yes) in each experiment. These plots are shown below.**

**As the plots show descriptively, recall error tended to decrease if participants fixated on the target during encoding, but there was not much of any added benefit if they additionally fixated on it during the RI. The only benefit of fixating on the target during the RI seems to be when participants had not fixated on it during encoding in Experiment 1B. Thus, these plots are generally consistent with the reported results: There is not much benefit of RI fixations, except in Experiment 1B, and the plot suggests that the benefit we observed in Experiment 1B may be more specific to targets that were not also fixated during encoding.**

**Even though this is just a descriptive and cursory consideration at this stage, we hope that adding it to our analysis scripts on the OSF will inspire future researchers to take up this interesting question in a more principled way than we have here.**

------

**R1.21:**

Exp2 RI shows much lower preference for fixating on memoranda locations when placeholders absent vs other experiments. Is this effect due to the memoranda being presented in a smaller segment of the screen in Exp2 than Exp1? Perhaps there is an effect of the eyes wandering away from the center of the screen that accounts for apparent looking-at-nothing in Exp1 that could be examined with Exp2 data. This could be done by dividing the screen into four sections: stimulus locations used on this trial, stimulus locations not used on this trial (i.e., the stimulus ring around the center, not including this trial’s quadrant), center, and other. Presumably, however, stimulus locations not used on this trial would count as “other” in Fig2. If there was an eyes wandering from center effect, “other” should have a higher fixation rate during RI than was observed. So, there does not appear to be an eye wandering effect, but where do participants eyes go during retention in Exp. 2? It seems like fixation rate is just lower in Exp2 than Exp1A and 1B. Maybe this has to do with fixation rate vs fixation duration? There is no need to address these question in revision, but it is a curious finding.

**Response:**

**This is an interesting observation, but the likely explanation is simply that we used different eye trackers that sampled eye movements at different rates between Experiment 1 (1000 Hz) and Experiment 2 (150 Hz) because they took place in different labs. Thus, it is not advisable to compare the fixation rate between the two experiments given that there were generally more fixations recorded overall, and we have made this more explicit on page 17. A great way to further demonstrate this is by focusing on the encoding panels of the figure – one can see that there were fewer fixations to the memoranda in Experiment 2 during encoding than Experiment 1, but this is not because participants were looking at the memoranda less, but it is more likely a sampling rate difference between the experiments. Finally, it is correct that stimulus locations on a particular trial of Experiment 2 would be classified as “other”, and we have made that more explicit on page 14.**

**Reviewer 2’s comments**

**R2.1:**

This study presents two experiments of which the goal is to verify whether eye movements can be used as an online measure of refreshing. Participants had to remember arrays of n colors. Experiment 1 used spontaneous and instructed refreshing during the retention interval, testing one item in the end. Experiment 2 used trials with and without placeholders to guide fixations during the retention interval, and tested all of the memory items.

The conclusion of the study is less clear to me, I have difficulty giving a clear take home message. On the one hand the authors state that “fixations during instructed but not spontaneous refreshing conditions account for additional variance in recall precision.” But then also: “Eye movements, however, do not seem suitable as an online measure of refreshing”.

IF (!) I understood everything correctly, the conclusion should just be that eye movements do not seem suitable as an online measure of refreshing (see comment below p 20-21). Is that correct?

**Response:**

**We agree that these last sentences of the abstract are a bit ambiguous – we have clarified what we mean by this sentence by now explicitly stating that, “Fixations under spontaneous conditions had no credible effect on recall precision, whereas the beneficial effect of fixations under instructed refreshing conditions may indicate situations in which cues were disregarded. Consequently, we conclude that eye movements do not seem suitable as an online measure of refreshing” (see page 2).**

------

**R2.2:**

The abstract is written very clear (except for the conclusion). The research question is very relevant and immediately captured my interest! And even though the answer may be negative in the end, at least we have an answer to a pending question!

**Response:**

**Thank you very much for these positive comments. We fully agree that even a negative answer will be important and informative for the field.**

------

**R2.3:**

I found it difficult to follow the manuscript at several moment. I wonder if it might be easier to split the experiment in experiment 1 and then Experiment 2, and for the analysis clearly split those for experiment 1A and 1B. I was often confused as Experiment 1A and 2 are often taken together for the analysis but this is not always clearly indicated so this led to confusion for me on several moments (whether the paragraph concerned only Experiment 1A or Experiment 1 as a whole). I think it may be easier to start by Experiment 1, show its shortcomings and then state in what way Experiment 2 can answer those.

**Response:**

**The revised manuscript now more clearly separates the different analyses of each experiment into separate subsections within the three different research questions. Furthermore, we have reiterated throughout each subsection which experiment and measure is being assessed, with stronger introductions to each analysis and with clearer reference to the figures as well. These changes can be seen throughout the results section on pages 17-23. We hope this makes it clear which experiment and analysis are considered while also retaining how those experiments/analyses address each research question.**

------

**R2.4:**

P 13 There is only 14% of the fixations qualified as looking toward the eventually tested item. It is stated that for that reason Experiment 2 was done. Is there an explicit reason that the instructed refreshing condition is not replicated? As this one seemed potentially to lead to interesting results?

**Response:**

**Experiment 1B sufficiently addressed our research question about instructed refreshing, whereas there were still lingering issues about spontaneous refreshing that could not addressed by Experiment 1A. Thus, we focused on addressing only those remaining issues in Experiment 2. We have made this more explicit on page 5.**

------

**R2.5:**

P 16 Is there a reason why the fixation rate would be greater during the 2.5 then during the 4 s interval ( and would the fixation rate be similar during the first 2.5 seconds? And then after 2.5 seconds there would be less fixations)

**Response:**

**It is not immediately clear to why fixations decrease over the RI, but this has been observed in other studies as well (see Souza et al., 2020) even in conditions in which fixations did predict better memory. For example, Souza et al. (2020) observed that fixations toward target locations in a spatial WM task predicted better recall, and more so when placeholders were onscreen. Yet similarly to what was observed here, fixation rate decreased when comparing a RI of 1s vs. 4s. We can only speculate about the reasons for that, but it might be due to the novelty declining since there was nothing new to see. We have added this point to page 18.**

**Given that we additionally assessed fixation duration in response to another comment (see R1.9 and E.7), we could determine whether fixations perhaps lingered longer on the dot locations when there was more time to do so, resulting in a lower fixation rate in the 4s condition during the RI. However, as can be seen in this figure appended again below, there are no differences in proportion of fixation duration between the RI conditions in either experiment. Given the subsequent analyses in the manuscript that go onto indicate these may have been largely random, ineffective fixations, we do not interpret the three-way interactions very strongly.**

------

**R2.6:**

P 20-21: “Follow-up analyses showed that the effect of fixations was not credible for 2 refreshing cues (estimate = 0.06 [-0.20, 0.33]), positive and credible for 1 refreshing cue (estimate = 0.50 [0.11, 0.90]), and positive but not credible for 0 refreshing cues (estimate = 0.27 [-0.22, 0.77]; see Figure 5C). All the remaining interactions were not credible. In summary, these results indicate that looking-at-nothing behavior under instructed refreshing conditions is functional to WM, and, interestingly, not redundant with the impact of refreshing cues. Fixations appeared to capture additional variance in recall precision that tended to be more pronounced with fewer refreshing cues.”

I have my doubts about this latter conclusion. The authors say that the effect is positive for 1 refreshing cue. That seems good news and in line with the conclusion that looking- at -nothing behavior under instructed refreshing is functional to WM. However, the fact that the effect was not credible for 2 refreshing cues nullifies this conclusion to me. Having 2 refreshing cues should at least have the same effect as 1 refreshing cue (and then maybe more but not per se). Can the authors elaborate more on their point of view as to why the evidence supports this conclusion?

**Response:**

**This has now been revised in response to other comments on this same issue (E.1 and R1.6). In short, we had conducted follow-up analyses in brms to decompose this interaction which led to results that were not quite right. The revised manuscript and the results on the OSF are in line with this conclusion, such that the effect of fixations was credible for 0 refreshing cues, smaller but still credible for 1 refreshing cue, and not credible for 2 refreshing cues (see page 23).**

------

**R2.7:**

In general, I guess the manuscript has a potential that is larger than it shows right now if it would be rewritten in a more straightforward way and with a clear conclusion.

**Response:**

**Thank you for your positive feedback and constructive comments – we hope that the revised manuscript better reflects the extent of its potential.**
